# ML241 Antagonizes ERK 1/2 Activation and Inhibits Rotavirus Proliferation

**DOI:** 10.3390/v16040623

**Published:** 2024-04-17

**Authors:** Jinlan Wang, Xiaoqing Hu, Jinyuan Wu, Xiaochen Lin, Rong Chen, Chenxing Lu, Xiaopeng Song, Qingmei Leng, Yan Li, Xiangjing Kuang, Jinmei Li, Lida Yao, Xianqiong Tang, Jun Ye, Guangming Zhang, Maosheng Sun, Yan Zhou, Hongjun Li

**Affiliations:** Institute of Medical Biology, Chinese Academy of Medical Science & Peking Union Medical College, Yunnan Key Laboratory of Vaccine Research and Development on Severe Infectious Disease, Kunming 650118, China; lanlingyu@student.pumc.edu.cn (J.W.); huxiaoqing@imbcams.com.cn (X.H.); wujinyuan@imbcams.com.cn (J.W.); linxiaochen@imbcams.com.cn (X.L.); chenrong@imbcams.com.cn (R.C.); luchenxing@student.pumc.edu.cn (C.L.); igtheshy131@gmail.com (X.S.); lqm212855240@163.com (Q.L.); yjlz2314@163.com (Y.L.); kuangxiangjun@imbcams.com.cn (X.K.); lijinmei917@163.com (J.L.); adayao0926@163.com (L.Y.); tangxq8859@163.com (X.T.); yejun@imbcams.com.cn (J.Y.); zhangguangming@imbcams.com.cn (G.Z.); sunmaosheng@imbcams.com.cn (M.S.)

**Keywords:** rotavirus, ML241 (hydrochloride), MAPK signaling pathway, ERK1/2, NF-κB

## Abstract

Rotavirus (RV) is the main pathogen that causes severe diarrhea in infants and children under 5 years of age. No specific antiviral therapies or licensed anti-rotavirus drugs are available. It is crucial to develop effective and low-toxicity anti-rotavirus small-molecule drugs that act on novel host targets. In this study, a new anti-rotavirus compound was selected by ELISA, and cell activity was detected from 453 small-molecule compounds. The anti-RV effects and underlying mechanisms of the screened compounds were explored. In vitro experimental results showed that the small-molecule compound ML241 has a good effect on inhibiting rotavirus proliferation and has low cytotoxicity during the virus adsorption, cell entry, and replication stages. In addition to its in vitro effects, ML241 also exerted anti-RV effects in a suckling mouse model. Transcriptome sequencing was performed after adding ML241 to cells infected with RV. The results showed that ML241 inhibited the phosphorylation of ERK1/2 in the MAPK signaling pathway, thereby inhibiting IκBα, activating the NF-κB signaling pathway, and playing an anti-RV role. These results provide an experimental basis for specific anti-RV small-molecule compounds or compound combinations, which is beneficial for the development of anti-RV drugs.

## 1. Introduction

Rotavirus (RV) is the main pathogen that causes severe diarrhea in infants and children under 5 years of age, with infection causing approximately 130,000 deaths annually [1]. Although licensed rotavirus vaccines provide more than 50% protection against rotavirus infection [2], currently, there are no specific antiviral treatments. The available treatments for the etiology of rotavirus-induced gastroenteritis are mainly symptomatic treatments and the correction of water and electrolyte imbalances using oral solutions to prevent or treat dehydration to reduce the duration and severity of diarrheal episodes [3,4]. Therefore, the control of rotavirus-induced gastroenteritis is of great importance for targeted interventions, such as the development of new small-molecule compound drugs to prevent and treat rotavirus-induced gastroenteritis.

Research on anti-RV drugs has shown that 2′-C-methylnucleoside [2CMC], 2′-C-methyladenosine [2CMA], 2′-C-methylguanosine [2CMG], and 7-deaza-2′-C-methyladenosine [7DMA] can inhibit rotavirus, sapoviruses, and norovirus by inhibiting viral genome transcription [5]. Genipin, isolated from jasmine flowers, inhibits human rotavirus Wa strain and simian rotavirus SA-11 strain in vitro by inhibiting two different stages of the viral replication cycle: attachment and penetration (early stage) in pre-treatment and assembly and release (late stage) in post-treatment [6]. Deoxyshikonin can inhibit rotavirus replication by inducing low SIRT1, ac-Foxo1, Rab7, and VP6 protein levels, low RV titers, low autophagy, and oxidative stress [7]. The antiviral effect of Portulaca oleracea L. polysaccharide (POL-P), an active component of Portulaca oleracea L(POL), inhibits rotavirus replication by upregulating the expression of IFN-α [8]. Inhibitors of dihydroorotate dehydrogenase (the rate-limiting enzyme for de novo pyrimidine synthesis) (BQR) can resist rotavirus infection by inhibiting pyrimidine biosynthesis in cells and intestinal organoids [9]. The small-molecule compound ML-60218 is an RNA polymerase III inhibitor that inhibits viral replication by destroying the viral cytoplasmic structure (viroplasm) [10]. The organ transplant immunosuppressive drug, 6-thioguanine (6-TG), inhibits rotavirus replication in Caco-2 cells and HIEs by interacting with the cellular drug target Rac1. Thiazolactones inhibit viral proliferation by inhibiting the formation of viral cytoplasmic structures (viroplasms) [11]. Metformin hydrochloride significantly inhibited the expression of rotavirus mRNA and protein in Caco-2 cells, small intestinal organoids, and lactational mouse models [12]. Dyngo-4a can inhibit rotavirus infection in vivo and in vitro by affecting the formation of dynamin-2 oligomers [13]. These studies screened compounds from animal sources or laboratory rotavirus strains, explored the compounds’ mechanisms of action on RV in vivo and in vitro, and provided treatment strategies for clinical symptoms caused by RV infection.

To find effective and low-toxicity anti-rotavirus small-molecule drugs, a wild human rotavirus ZTR-68 strain was used for drug screening from 453 small-molecule compounds. The anti-rotavirus activity was tested using an Enzyme-Linked Immunosorbent Assay (ELISA). The role of selected compounds in the adsorption, cell entry, and replication stages of the virus was studied using NSP3 real-time quantitative PCR (RT-qPCR) for rotaviral NSP3 and Western blot for rotaviral VP7 and NSP3. The antiviral mechanism of the compound was analyzed through transcriptome sequencing and WB, and the signaling pathway through which the compound exerted its inhibitory effect on rotavirus replication was determined. Suckling mice were used as a model to study the in vivo anti-RV effects of the compounds. In summary, this study discovered a small-molecule compound that effectively inhibits rotavirus replication and the mechanism underlying this.

## 2. Materials and Methods

### 2.1. Cell Culture

African green monkey embryonic kidney cells (MA104) were provided by the Molecular Biology Department of the Institute of Medical Biology, Chinese Academy of Medical Sciences, and Peking Union Medical College. The cells were cultured at 37 °C in a 5% carbon dioxide atmosphere in Dulbecco’s Modified Eagle Medium (DMEM) containing 10% fetal bovine serum (FBS) and 1% double antibiotics (100 U/mL penicillin and 100 μg/mL streptomycin).

### 2.2. Rotavirus Amplification and Titer Determination

The genotype of rotavirus ZTR-68 is G1P [8], and the genotype of the SA11 strain is G3P [2]. They were isolated and preserved at the Molecular Biology Laboratory of the Institute of Medical Biology, Chinese Academy of Medical Sciences, and Peking Union Medical College. The virus titer was determined using the Kaerbar method with the following formula: LgCCID50 = Xm-1/2d + d∙∑pi/100. The cutoff value was 0.105. The titer of the ZTR-68 strain was found to be 7.5 LgCCID_50_/mL, and that of the SA11 strain was 7.0 LgCCID_50_/mL. The titer of the Wa strain was 6.5 LgCCID_50_/mL and that of the Gottfried strain was 7.9 LgCCID_50_/mL. To analyze whether ML241 affects the entry of RV into its host cells, the viruses were treated with ultraviolet irradiation at 220 nm (UV dose 22.5 mJ/cm^2^). Irradiation with 220 nm of UV destroyed the nucleic acids in the viruses; therefore, RNA replication and protein translation could not be performed. However, this process did not affect virus entry into host cells or RNA release [14].

### 2.3. Enzyme-Linked Immunosorbent Assay

MA104 cells were transferred to a 96-well culture plate. When the cells grew to form a dense monolayer, the RV was activated with 20 μg/mL acetylase and 600 μg/mL CaCl_2_. The multiplicity of virus infection was MOI = 0.1, and different concentrations were immediately added. After complete CPE was observed in the virus control group, the culture was frozen and thawed three times. While conducting the large-scale screening of the anti-RV small-molecule compounds and after exploring the optimal viral load of the reference virus to determine the optimal MOI through multiple preliminary experiments, we chose to use ELISA quantitative detection methods to screen the compounds [15,16,17,18]. A total of 453 small-molecule compounds were screened for rotavirus proliferation using an ELISA. The original solution of the inactivated rotavirus vaccine was used as the standard. The antigen content was 1236 EU (ELISA unit, EU)/mL. A standard curve was constructed using a 2-fold dilution of 12 standard gradients. A 50-fold-diluted standard was used as the internal reference. The OD_450–650_ value was read using a microplate reader. GraphPad Prism 9.3.1 software was used to run the sigmoidal 4PL; the antigen content of the virus that proliferated was used as the virus control, and the virus that did not proliferate was used as the blank control to calculate the inhibition rate of the virus by the small-molecule compound. The calculation formula was as follows: inhibition rate (%) = (A_compound group_ − A_virus group_)/(A_blank group_ − A_virus group_) × 100%. In this experiment, a purified goat anti-rotavirus G1P [8] antibody (batch number: RVAB2020101), preserved by the Institute of Medical Biology, Chinese Academy of Medical Sciences, was used as the primary antibody in the ELISA experiment, and the secondary antibody was an HRP-labeled goat anti-mouse purified antibody (batch number: RVAB2020101H).

### 2.4. Cell Viability Determination

A Cell Counting Kit-8 (CCK8) kit (CA1210, Solarbio, Beijing, China) was used to measure the toxic effects of small-molecule compounds on cell proliferation. After the small-molecule compounds were used for treatment with different concentration gradients for 48 h, 10% CCK8 solution was added, and the absorbance at 450 nm was measured using a microplate reader. The cell group without small-molecule compounds was used as a control, and the group without cultured cells was used as a blank control. Cell viability was calculated using the following formula: cell viability (%) = (A_compound group_ − A_blank group)_/(A_cell group_ − A_blank group_) × 100%. Then, the toxicity of small-molecule compounds to cell proliferation was determined.

### 2.5. Immunofluorescence

We first transferred MA104 cells to a 12-well culture plate. When the cells grew to a dense monolayer, the RV was activated with 20 μg/mL acetylase and 600 μg/mL CaCl_2_. The multiplicity of virus infection was MOI = 0.1, and a combined ELISA experiment was performed. Compounds with the optimal concentration measured in the CCK8 experiment were incubated for 16 h at 37 °C and 5% CO_2_ and then taken out for immunofluorescence experiments. In this experiment, 4% paraformaldehyde containing 0.2% Triton (batch number RVAB2019101) was used. In this experiment, a purified goat anti-rotavirus antibody (batch number: RVAB2020101), preserved by the Institute of Medical Biology, Chinese Academy of Medical Sciences, was used as the primary antibody in the immunofluorescence experiment. The secondary antibody used was a fluorescein isothiocyanate (FITC)-labeled rabbit anti-goat antibody (Cat. No. 305-095-003, Jackson Immune Research, United States). 4′,6-diamidino-2-Phenylindole (DAPI) (Cat. No. C1005, Beyotime, Zhengzhou, China) was used to stain the cell nuclei, and then we observed and collected images using a fluorescence inverted microscope.

### 2.6. Real-Time Fluorescence Quantitative PCR

The viral genomic dsRNA was detected using RT-qPCR [19]. After extracting the viral genomic RNA, we used a HiScript^®^ II One Step qRT-PCR SYBR Green Kit (Q222, Novozant, Nanjing, China) to detect the Ct value of the genomic dsRNA, which was also determined using RT-qPCR. In addition, to obtain a standard curve, the genomic dsRNA, which was used as the standard, was diluted in a gradient and the copy number of genomic dsRNA was detected using RT-qPCR. Finally, the number of virus copies was calculated based on the standard curve. We designed specific primers and probes targeting the highly conserved region of the NSP3 gene (Table 1).

The differentially expressed genes of the MA104 cells were also detected using RT-qPCR. We added the RVs (MOI = 0.1) and ML241 (20μM) to MA104 cells. After 20 h of infection, the cells were washed twice with PBS and then the RNA of the MA104 cells was extracted using trizol. After extracting the RNA from the MA104 cells, we used a HiScript^®^ II One Step qRT-PCR SYBR Green Kit (Q221, Novozant, Nanjing, China) to detect the differentially expressed genes of the MA104 cells using RT-qPCR. We measured the relative expression level of the target gene using the gene of β-actin as the internal reference gene.

### 2.7. Western Blotting

Approximately 20 μM ML241 and RV were added to MA104 cells grown in a dense monolayer in sequence, with MOI = 0.1. After 20 h of incubation, the cell surface was gently washed twice with PBS, and a high-efficiency RIPA cell lysis buffer (R0010, Solarbio) was used to extract the total cell proteins. The bicinchoninic acid (BCA) protein concentration determination kit (P0012, Beyotime, Zhengzhou, China) was used to determine the protein concentration, and then Western blotting was performed.

### 2.8. Animal Experiments

The experimental protocol was approved (DWLL202208006) by the Experimental Animal Welfare Ethics Committee of the Institute of Medical Biology within the Chinese Academy of Medical Sciences (Beijing, China). The SA11 strain was used to establish a suckling mouse model to evaluate the in vivo anti-RV effects of ML241. The groups are listed in Table 2. The diarrhea score was based on the scoring rules for diarrhea in suckling rats proposed by BOSHUTZENJA et al. [20]. Diarrhea in suckling rats was scored from 0 to 4 based on the color, hardness, and quantity of feces. The score for no feces discharged is 0 points; the score for brown formed stool is 1 point; the score for brown soft stool is 2 points; the score for yellow soft stool is 3 points; the score for yellow watery stool is 4 points; and the score for perianal fecal contamination is 4 points. A score greater than 2 points was considered an indication of diarrhea.

### 2.9. HE Staining Experiment for Small Intestinal Tissue

The small intestinal tissue of neonatal mice was dissected and immediately placed in a tissue fixative (Cat. No.: G1101, Servicebio, Wuhan, China), fixed for 24 h, dehydrated, soaked in wax, embedded in paraffin, and then cooled on a −20 °C freezing table. Paraffin sections were 4 μm thick. The paraffin sections were then dewaxed, covered with water, stained with hematoxylin and eosin in sequence, dehydrated, and mounted for microscopic observation to collect images.

### 2.10. Transmission Electron Microscopy Experiment of Small Intestinal Tissue

The small intestinal tissue of neonatal mice was dissected to a size of 1 mm^3^ and stored in an electron microscope fixative (Cat. No.: G1102; Servicebio, Wuhan, China) at 4 °C. Then, 1% osmic acid was prepared in 0.1 M phosphate buffer PB (PH 7.4) to protect the samples from light and fixed for 2 h. After that, they were rinsed with 0.1 M phosphate buffer (PB) (pH 7.4) and dehydrated at 24 °C. After permeation, embedding, polymerization, and staining, transmission electron microscopy was used to observe the small intestinal tissue and to collect images.

### 2.11. Statistical Analyses

GraphPad Prism 9.3.1 (GraphPad, La Jolla, CA, USA) was used for data analyses and mapping. Experimental results are expressed as the geometric mean ± standard error. Between-group differences were analyzed using the two-tailed Student’s *t*-test or Prapey multiple comparison test. *p* < 0.05 was considered significant.

## 3. Results

### 3.1. Screening of Anti-RV Small-Molecule Compounds

It is critical to determine the viral infection dose for screening compounds. Through pre-experimental screening, the small-molecule compounds that resisted the proliferation of the rotavirus ZTR-68 strain were screened from a library of 453 small-molecule compounds, and the optimal viral infection dose MOI = 0.1 was found. After determining the amount of infectious virus, five concentration gradients of 10 μM, 1 μM, 100 nM, 10 nM, and 1 nM were set according to the recommended concentrations of the compound library. The ELISA method was used to determine the effect of small-molecule compounds on inhibiting rotavirus proliferation. The 126 compounds that could significantly inhibit the proliferation of the rotavirus ZTR-68 strain at a concentration of approximately 10 μM were further tested through cell toxicity testing. Compounds with high toxicity to MA104 cells were removed, and the remaining five compounds with relatively low toxicity were subjected to a second round of screening.

**Figure 1:**
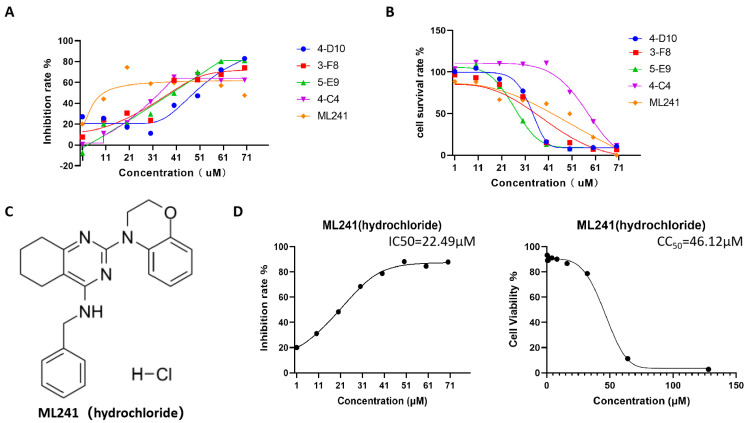
Screening of anti-RV small-molecule compounds. (**A**) ELISA was used to detect the inhibitory rate of five compounds against rotavirus. (**B**) CCK8 was used to measure the toxic effects of the five compounds on the cells. (**C**) The structural formula of ML241 (hydrochloride). (**D**) Half of the inhibitory rate of ML241 against rotavirus and half of its toxic effect on MA104 cells.

Furthermore, five small-molecule compounds, namely 4-D10, 3-F8, 5-E9, 4-C4, and ML241, were used in experiments on the inhibitory effect on rotavirus proliferation and cell proliferation (survival). Toxicity testing was undertaken with eight concentration gradients of 70, 60, 50, 40, 30, 20, 10, and 1 μM. The results showed that at a concentration of 20 μM, compared with the other four compounds, ML241 had the best inhibitory effect on rotavirus (Figure 1A) and was less toxic to MA104 cells, the host cells of rotavirus (Figure 1B). Based on these results, ML241 was screened out. The molecular structural formula of ML241 is C23H25CIN4O (Figure 1C). It was calculated and measured that the half-toxic concentration of the ML241 drug was CC50 = 45.42 ± 1.03μM, the half inhibitory concentration of the drug IC50 = 24.38 ± 4.33 μM, and SI (CC50/IC50) = 1.93 ± 0.36 (Figure 1D).

### 3.2. In Vitro Effects of ML241 on Rotavirus

**Figure 2:**
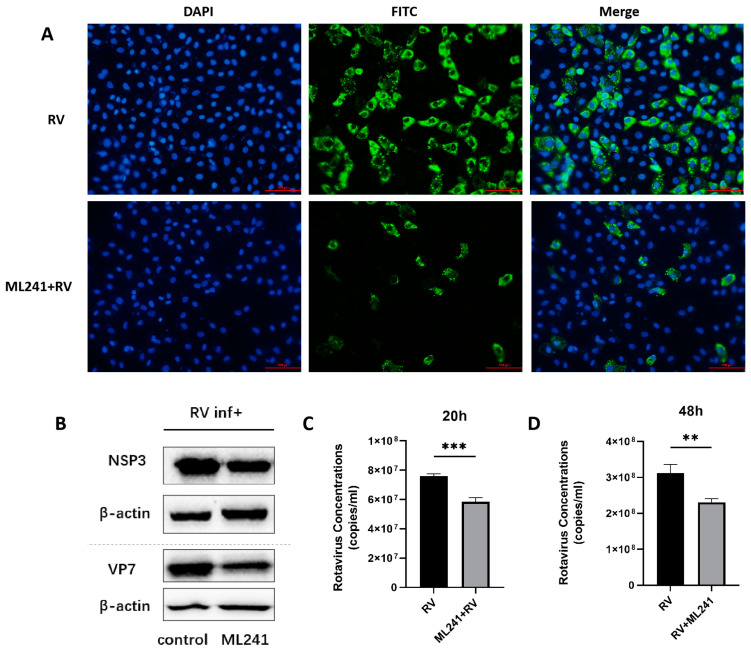
In vitro effects of ML241 on rotavirus. (**A**) Immunofluorescence experiments verified the inhibitory effect of ML241 on RV. (**B**) Western blotting was used to detect the expression of NSP3 and VP7 after adding ML241 for 20 h. (**C**) The RV copy number was measured by RT-qPCR using ML241 after 20 h of infection. (**D**) The RV copy number was measured by RT-qPCR using ML241 after 48 h of infection. Data are presented as mean ± SD. Significant differences were determined by an unpaired *t* test (** *p* < 0.01, *** *p* < 0.001).

To analyze the effect of the small-molecule compound ML241 on RV, immunofluorescence, R-qPCR, and WB were used to detect viral protein expression and viral replication 20 h after the addition of the drug and virus. The results showed that, compared with the control group, the addition of ML241 inhibited the expression of viral proteins and viral replication (Figure 2A–C).

**Figure 3:**
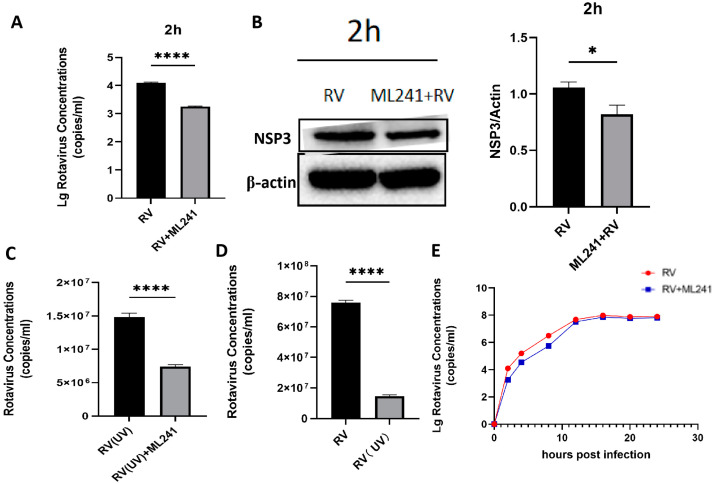
In vitro inhibitory effects of ML241 on rotavirus. (**A**) The RV copy number was measured by RT-qPCR using ML241 after 2 h of infection. (**B**) The expression of NSP3 was detected after adding ML241 for 2 h by a Western blotting experiment and the value of NSP3/β-actin was 1.30 ± 0.18. (**C**) RT-qPCR detection, with ML241, increased the rotavirus (RV) copy number following 20 h of UV irradiation. (**D**) RT-qPCR detects the copy number of RV and UV-irradiated RV at 20 h. (**E**) RT-qPCR is used to detect the copy number of RV at different times after the addition of ML241. Data are presented as mean ± SD. Significant differences were determined by an unpaired *t* test (* *p* < 0.05, **** *p* < 0.0001).

To analyze whether ML241 affected the process by which RV entered a cell, the virus copy number and NSP3 protein expression 2 h after viral infection were detected using RT-qPCR and WB. The results showed that after adding ML241 for 2 h, the virus copy number decreased (Figure 3A), and the expression of the NSP3 protein decreased (Figure 3B). To analyze whether ML241 affected the process of RV entry into its host cells, the viruses were treated with ultraviolet irradiation at 220 nm (UV dose 22.5 mJ/cm^2^) to disrupt their nucleic acids and prevent them from RNA replication and protein translation. Compared with the RV group without UV irradiation, the NSP3 copy number was significantly reduced after UV irradiation (Figure 3C). After adding ML241 to the UV-irradiated RV group, NSP3 also decreased compared with the UV-irradiated RV group. This decrease (Figure 3D) suggests that ML241 affected the process of RV entry into its host cells. RT-qPCR was used to detect the NSP3 copy number at different time points, and it was found that ML241 had a significant inhibitory effect at the early stage of RV infection (Figure 3E), and its inhibition of rotavirus proliferation was still statistically significant until 48 h (Figure 2D).

**Figure 4:**
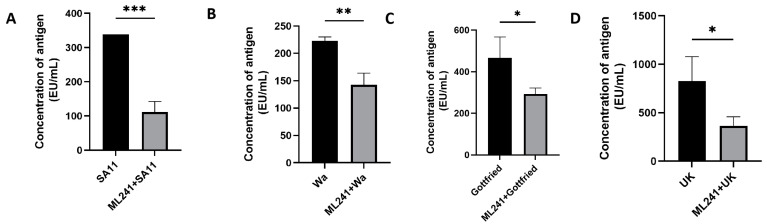
In vitro inhibitory effects of ML241 on rotavirus SA11, Wa, Gottfried, and UK strains. The anti-rotavirus activity was tested by Enzyme-Linked Immunosorbent Assay (ELISA). (**A**) In vitro inhibitory effects of ML241 on rotavirus SA11 strains. (**B**) In vitro inhibitory effects of ML241 on rotavirus Wa strains. (**C**) In vitro inhibitory effects of ML241 on rotavirus Gottfried strains. (**D**) In vitro inhibitory effects of ML241 on rotavirus UK strains. Data are presented as mean ± SD. Significant differences were determined by an unpaired *t* test (* *p* < 0.05, ** *p* < 0.01, *** *p* < 0.001).

To verify the inhibitory effect of ML241 on other rotavirus strains, we measured the amount of antigen in different rotavirus strains after the administration of ML241 by ELISA. The results showed that ML241 had inhibitory effects on the RV of SA11, UK, Wa, and Gottfried strains (Figure 4).

### 3.3. In Vivo Effects of ML241 on Rotavirus

**Figure 5:**
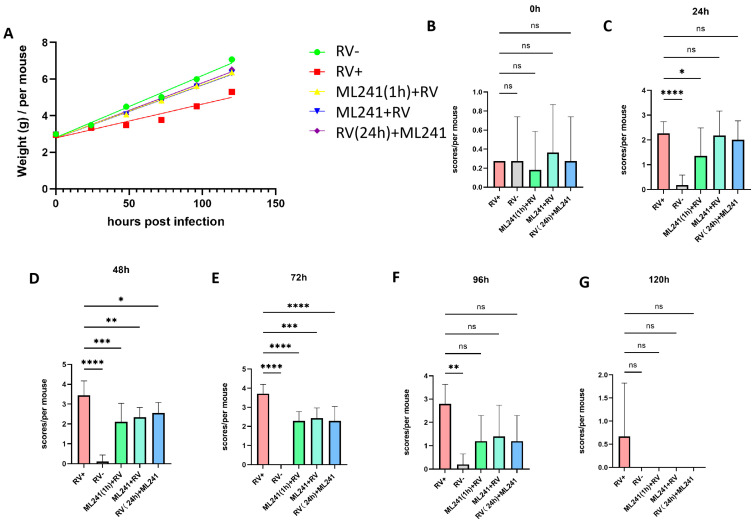
In vivo effects of ML241 on rotavirus. (**A**) Body weights of the suckling mice in each group. (**B**) Diarrhea scores of suckling mice in each group before challenge. (**C**) Diarrhea scores of suckling mice in each group 24 h after challenge. (**D**) Diarrhea scores of suckling mice in each group 48 h after challenge. (**E**) Diarrhea scores of suckling mice in each group 72 h after challenge. (**F**) Diarrhea scores of suckling mice in each group 96 h after challenge. (**G**) Diarrhea scores of suckling mice in each group 120 h after challenge. Data are presented as mean ± SD. Significant differences were determined by an unpaired *t* test (ns *p* > 0.05, * *p* < 0.05, ** *p* < 0.01, *** *p* < 0.001, **** *p* < 0.001).

A 5-day-old BALB/c suckling mouse diarrhea model was established to test the inhibitory effect of ML241 on rotavirus in vivo. The grouping information is presented in Table 1. The body weight of the suckling mice was measured before and 24 h, 48 h, 72 h, 96 h, and 120 h after challenge with the SA11 strain of RV, and their diarrhea scores were calculated. The results showed that, compared with the normal control group, the weight gain of mice in the SA11 challenge group (model group) was slower, whereas the weight gain of the ML241-treated group was significantly higher than that of the model group (Figure 5A). Before the challenge, there was no statistical difference in diarrhea scores between the groups (Figure 5B). Twenty-four hours after the challenge, the diarrhea score of the RV model group was significantly higher than that of the normal control group, indicating that a suckling mouse diarrhea model of RV infection was successfully created (Figure 5C). After 48 h, compared to the model group, the scores of compound groups decreased, and there were significant differences in all scores (Figure 5D), with the most obvious being observed at 72 h (Figure 5E). There was no difference between the groups at 96 h and 120 h (Figure 5F,G). The results showed that prevention or treatment with ML241 can reduce the degree of diarrhea in suckling mice.

**Figure 6:**
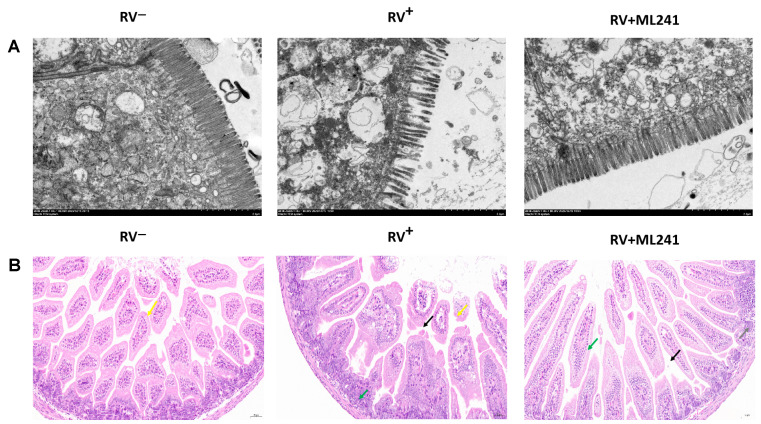
In vivo effects of ML241 on rotavirus. (**A**) Electron microscopic observation of small intestinal lesions in the different treatment groups. (**B**) HE staining was used to observe small intestinal lesions in the different treatment groups.

Two suckling mice were randomly dissected at 24 h, 48 h, 72 h, 96 h, and 120 h after the challenge, and their hearts, livers, spleens, lungs, kidneys, stomachs, and intestines were collected. Electron microscopy results at 72 h showed (Figure 6A) that the microvilli in the small intestine of the unchallenged group (normal control group) of suckling mice were densely arranged and neatly structured. The small intestinal microvilli of the challenge group (model group) were shortened, loosely arranged, and disordered; the basal layer was loose; the small intestinal villi were severely vacuolated; and in some places, they even fell off and caused gaps. The microvilli in the small intestine of suckling mice in the ML241 intervention and challenge groups were slightly shortened and loosely arranged; however, the situation was significantly better than that in the non-intervention challenge group.

HE staining of the small intestinal tissue of suckling mice (Figure 6B) showed that the small intestinal tissue of the unchallenged mice (normal control group) had a normal length of intestinal villi (yellow arrow) and abundant intestinal glands in the lamina propria, which were densely arranged and of a short tubular shape. The structure of the muscle layer was clear and the muscle cells were regularly arranged. In the challenge group (model group), the intestinal villous epithelium was occasionally lost in the small intestinal tissue of the suckling mice (yellow arrow), a small amount of intestinal villous epithelium was separated from the lamina propria (black arrow), the gap was widened, and the intestinal glands in the lamina propria were numerous and densely arranged. A short tubular shape was observed, with occasional scattered granulocytic infiltration (green arrow). The small intestinal tissue of the ML241 intervention group showed long intestinal villi, abundant intestinal villi, and an intact intestinal villus epithelium. Occasionally, the top of the intestinal villous epithelium separated from the lamina propria (black arrow), and the gap widened. There was a high number of intestinal glands in the lamina propria, which was large; it was in the shape of a short tube, with a small amount of vascular congestion (green arrow). Occasionally, a small focal accumulation of lymphocytes (gray arrow) was observed, along with a clear muscle layer structure and a regular arrangement of muscle cells. This shows that ML241 can significantly improve lesions in the small intestines of suckling mice, reduce diarrhea symptoms, and play a protective role in suckling mice.

### 3.4. ML241 Antagonizes ERK 1/2 Activation of the MAPK Signaling Pathway by RV and Inhibits Rotavirus Replication

**Figure 7:**
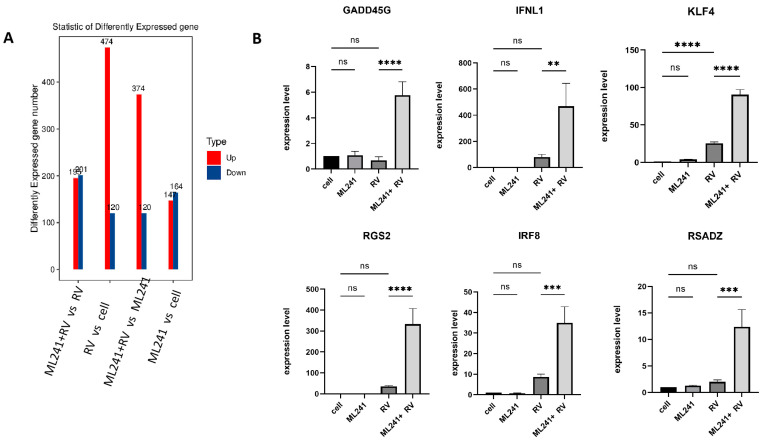
ML241 antagonizes ERK 1/2 activation of the MAPK signaling pathway via RV and inhibits rotavirus replication. (**A**) Number of differentially expressed genes in each group. (**B**) Relative expression of the differentially expressed genes in each group. (ns *p* > 0.05, ** *p* < 0.01, *** *p* < 0.001, **** *p* < 0.001).

**Figure 8:**
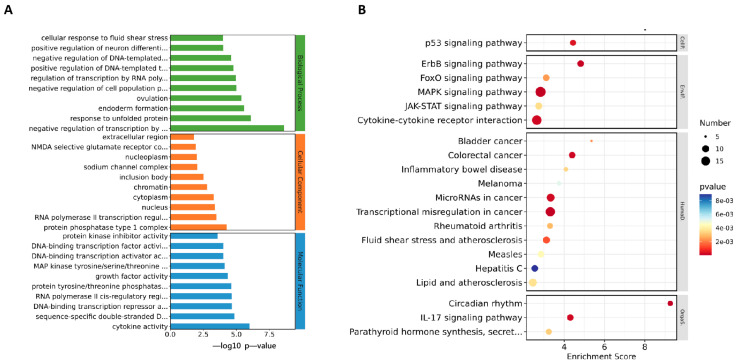
ML241 antagonizes ERK 1/2 activation of the MAPK signaling pathway via RV and inhibits rotavirus replication. (**A**) GO enrichment analysis of the top 30 genes with upregulated expression in ML241+RV vs. RV. (**B**) KEGG enrichment analysis of the top 20 genes with upregulated expression in ML241+RV vs. RV.

**Figure 9:**
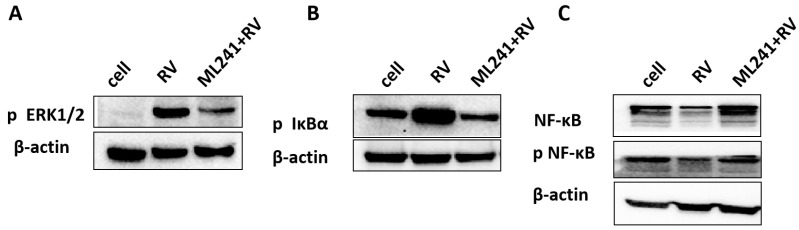
ML241 antagonizes ERK 1/2 activation of the MAPK signaling pathway via RV and inhibits rotavirus replication. (**A**) Western blotting was used to detect the expression of phosphorylated ERK1/2. (**B**) Western blotting was used to detect the expression of phosphorylated IκBα. (**C**) Western blotting was used to detect the expression of NF-κB and phosphorylated NF-κB.

To analyze the mechanism by which ML241 inhibits rotavirus replication, we performed transcriptome sequencing (RNA-seq) in three groups: cell, RV, and ML241 + RV. When using FC ≥ 2.0, compared with the RV group, there were 195 genes with upregulated expression and 201 genes with downregulated expression in the group of ML241+RV (Figure 7A). We performed RT-qPCR verification analysis on the top 15 genes with upregulated and downregulated expression (FC ≥ 2.0) in each group of sequencing results, and the results showed that they were consistent with the RV group; the addition of ML241 caused an increase in the expression of interferon- and interleukin-related transcription factors, such as GADD45G, IFNL1, IRF8, KLF4, RGS2, and RSADZ genes (Figure 7B). A gene ontology (GO) enrichment of differentially expressed genes was performed (Figure 8A). A set analysis showed that compared with the RV group, after adding ML241, the molecular function was mostly the activation of cytokines, the cellular composition was the activation of protein phosphatase type I complex, and the biological process was negative for transcription. The differential gene Encyclopedia of Genes and Genomes (KEGG) analysis showed (Figure 8B) that after the addition of ML241, the differential genes were mostly enriched in the MAPK signaling pathway. We speculated that the inhibitory effect of ML241 on RV proliferation may be mediated through the MAPK signaling pathway, which plays a role. After clarifying that the MAPK signaling pathway may be involved, we detected the key proteins in the MAPK signaling pathway through WB. The results showed that after adding RV, RV significantly activated the phosphorylation of extracellular signal-regulated kinase 1/2 (ERK1/2), and its downstream IκBα was significantly increased due to RV infection. When ML241 was added, ERK phosphorylation was weakened (Figure 9A), IκBα expression was reduced (Figure 9B), and NF-κB and pNF-κB were increased (Figure 9C).

## 4. Discussion

In this study, we screened 453 small-molecule compounds for anti-RV wild strain ZTR-68, which was isolated from humans using the ELISA assay. It was found that the small-molecule compound ML241 (hydrochloride) can inhibit the replication of the human rotavirus ZTR-68 strain, and the cytotoxicity test results showed that it has low toxicity to MA104 cells, which is the RV host cell. In vitro experiments showed that its inhibitory effect is particularly obvious in the early stages of RV infection, and it has inhibitory effects on the virus adsorption, cell entry, and replication stages. The antiviral mechanism of ML241 was analyzed through transcriptome sequencing and WB, and it was found that ML241 antagonizes ERK 1/2 activation and inhibits rotavirus proliferation. Using suckling mice as a model, we studied the in vivo anti-RV effect of ML241 and found that ML241 could reduce the severity of diarrhea in suckling mice and improve the degree of lesions in the small intestines of suckling mice. This study discovered a small-molecule compound that effectively inhibits rotavirus replication and studied its mechanism of action.

Small-molecule compounds are biologically active compounds with a molecular weight of less than 1000 Da (especially less than 500 Da). They can enter cells through the cell membrane, regulate targets in organelles, and carry out their corresponding biological functions. Compared with macromolecular compounds, small-molecule compounds have more advantages in terms of their targets (enzymes, ion channels, and receptors), their preparations, their costs, and patient compliance and have been widely used in virology, oncology, immunology, and neurology. Important research areas include biology, epigenetics, stem cells, organoids, apoptosis, ion channels, and signal transduction [21]. Antiviral small-molecule compounds mainly exert antiviral effects on virus adsorption, invasion, replication, assembly, and release by regulating host proteins or directly inhibiting viral proteins [22]. A variety of small-molecule drugs targeting SARS-CoV-2 have made breakthrough progress [23,24,25], and a variety of therapeutic drugs have entered Phase III clinical trials. To date, there are no specific antiviral therapies or marketed anti-rotavirus drugs against rotavirus. The development of anti-RV drug treatments can effectively prevent severe disease caused by viral infection, shorten the course of the disease, and alleviate symptoms.

ML241 (hydrochloride), CAS 2070015-13-1, chemical formula C23H25ClN4O, screened in this experiment, is an effective AAA ATPase p97 inhibitor [26]. The compound’s half-inhibitory concentration value is 100 nM and it is widely used in anti-tumor and anti-inflammatory research. AAA ATPase p97 maintains eukaryotic cell proteostasis by promoting the degradation of ubiquitinated proteins via the proteasome and the maturation of autophagosomes [27]. In this study, we found that ML241 inhibited RV proliferation in vivo and in vitro, especially at the early stages of RV infection. The in vivo experimental results showed that there was little difference in the therapeutic effect when ML241 was administered before and after viral infection.

Compared to the viral infection group, the differentially expressed genes were mainly clustered in the MAPK signaling pathway. Further analysis revealed that they mainly clustered in the mitogen-activated protein extracellular signal-regulated kinase/extracellular-regulated kinase (MEK/ERK) signaling cascade. This signaling pathway mediates a variety of processes, including cell adhesion, cell cycle progression, cell migration, cell survival, differentiation, inflammation, metabolism, proliferation, and transcription [28]. Studies have shown that RV promotes replication by regulating the MEK/ERK signaling pathway [29]. The RV-induced apoptosis observed in the early stages of infection is inhibited by RV nonstructural protein 1 through the activation of the PI3K/Akt and NF-κB pro-survival pathways [30,31,32]. Many viruses, including DNA and RNA viruses, utilize the MEK/ERK pathway to promote different stages of their life cycles [28]. In this study, after adding ML241, the phosphorylation of ERK in the MAPK signaling pathway was downregulated compared with that in the RV group. ML241 antagonizes the activation of ERK phosphorylation induced by RV and inhibits viral proliferation.

NSP1 is an RNA-binding protein [33] that evades the innate immune response and delays early apoptosis by inhibiting interferon (IFN) induction and activating the PI3K/Akt pathway [34,35]. NSP1 interacts with TRAF2 to inhibit interferon-induced atypical NF-κB activation and antagonizes virus-induced cytokine responses to promote virus reproduction [36]. In this study, we found that the downstream protein IκBα of ERK was inhibited. IκBα is an inhibitory protein in the nuclear factor-κB (NF-κB) signaling pathway [37,38]. Cells respond to inflammatory stimuli via the NF-κB signaling pathway. When IκBα is inhibited, the NF-κB signaling pathway is activated, which is consistent with our detection of the expression of numerous inflammation-related genes.

## 5. Conclusions

In conclusion, in this study, a compound that effectively inhibited the proliferation of the human rotavirus ZTR-68 strain at multiple replication stages was selected. The results of the signaling pathways analysis showed that ML241 could inhibit viral proliferation by antagonizing the activation of ERK in the MAPK pathway. Further, by using suckling mice as an animal model, the in vivo effects of ML241 were studied, and it was found that ML241 also has a good effect on inhibiting the proliferation of rotavirus in vivo and has a good protective and therapeutic effect on suckling mice. This study helps us to further understand the pathogenesis of rotavirus and provides research ideas for the development of drugs to inhibit rotavirus, which is of significance for the development of clinical drugs for the treatment of rotavirus diarrhea.

## Figures and Tables

**Table 1:** NSP3 primer and probe sequence.

	Name	Sequence
ZTR-68	Forward primer	ACCATCTACACATGACCCTC
	Reverse primsr	GGTCACATAACGCCCC
	TaqMan probe	FAM-ATGAGCACAATAGTTAAAAGCTAACACTGTCAA-TAMRA
SA11	Forward primer	GTTGTCATCTATGCATAACCCTC
	Reverse primsr	ACATAACGCCCCTATAGCCA
	TaqMan probe	FAM-ATGAGCACAATAGTTAAAAGCTAACACTGTCAA-TAMRA

**Table 2:** Grouping of suckling mice by gavage.

Group	Quantity	Virus (SA11) Dose	The Medicine Dose (mg/kg)	Frequency of Administration	Route of Administration
RV−	11	PBS (100 μL)	−	−	gavage
RV+	11	10^5^ pfu	−	−	gavage
ML241 (1 h) + RV	11	10^5^ pfu	20	QD	gavage
ML241 + RV	11	10^5^ pfu	20	QD	gavage
RV (24 h) + ML241	11	10^5^ pfu	20	QD	gavage

## Data Availability

Data are contained within the article.
